# Influence of Electric Current and Magnetic Flow on Firing Patterns of Pre-Bötzinger Complex Model

**DOI:** 10.1155/2021/6655933

**Published:** 2021-04-04

**Authors:** Wenchao Ji, Moutian Liu, Lixia Duan

**Affiliations:** School of Science, North China University of Technology, Beijing 100144, China

## Abstract

The dynamics of neuronal firing activity is vital for understanding the pathological respiratory rhythm. Studies on electrophysiology show that the magnetic flow is an essential factor that modulates the firing activities of neurons. By adding the magnetic flow to Butera's neuron model, we investigate how the electric current and magnetic flow influence neuronal activities under certain parametric restrictions. Using fast-slow decomposition and bifurcation analysis, we show that the variation of external electric current and magnetic flow leads to the change of the bistable structure of the system and hence results in the switch of neuronal firing pattern from one type to another.

## 1. Introduction

Breathing is an important physiological activity that is necessary for all mammals, including human beings, to sustain their lives. Experiments have shown that respiratory rhythms in the neonatal nervous system of mammals may be related to pacemaker neurons in the pre-Bötzinger complex (pre-BötC) [1, 2]. Solomon et al. found that the chemical stimulation of the pre-BötC in vivo manifests respiratory modulation consistent with a respiratory rhythm generator [3]. The phrenic motor activity evoked by chemical stimulation of the pre-BötC is affected and integrated through the regulation of the respiratory network driven by input from central and peripheral chemoreceptors [4]. Besides, Koizumi and Smith used real-time calcium activity imaging combined with whole-cell patch-clamp recording to analyze contributions of subthreshold conductances in the excitatory rhythm-generating network [5]. Negro et al. demonstrated that dendritic Ca^2+^ activates an inward current to electronically depolarize the soma, rather than propagating as a regenerative Ca^2+^ wave [6]. Research evidences underscored that respiratory rhythmogenesis may depend on dendritic burst-generating conductance activated in the context of network activity.

The mathematical framework of neuron electrophysiological models has been derived from the Hodgkin-Huxley (H-H) model established by Hodgkin and Huxley [7]. Based on the H-H model, Butera et al. created two mathematical models of inspiratory pacemaker neurons [8, 9] to simulate the respiratory rhythm generation of single as well as coupled pre-BötC neuron. Subsequent studies have shown that neurons within the pre-BötC have a persistent sodium (NaP) current and a calcium-activated nonspecific cationic (CAN) current. CAN current can be activated via second-messenger-mediated synaptic pathways [10, 11]. Driven by the experimental results, Toporikova and Butera proposed a pre-BötC (TB) model that encompasses both *I*_NaP_-dependent somatic bursting and *I*_CAN_-dependent dendritic bursting [12]. The model explains a number of conflicting experimental results, and it is able to generate a robust bursting rhythm, over a large range of parameters, with a frequency adjusted by neuromodulators. Later, Park and Rubin simplified the TB model and proposed a single-compartment model of a pre-BötC inspiratory neuron which is able to generate all major activity patterns seen in the two-compartment model [13]. Also, the neuronal models exhibit abundant dynamic characters such as the bifurcation phenomenon. Gu et al. demonstrated bifurcation and complex dynamic behaviors in biological experiments [14, 15].

An appropriate external stimulus can change the firing patterns of neurons. Much research in recent years has focused on the effects of electromagnetic radiation on neuronal behaviors [16–20]. Electromagnetic radiation can affect the dynamic characteristics of neurons, and electrical or electromagnetic stimulation can also be used to treat neurological diseases [21–23]. Recently, Song et al. designed a nonlinear circuit including an inductor, resistor, capacitor, and other electric devices. They found that the energy storage is dependent on the external forcing and the energy release is associated with the electric mode [24]. Lv and Ma also found that electromagnetic radiation can not only excite quiescent neurons but also suppress the electrical activities in the improved three-variable Hindmarsh-Rose model [25]. Duan et al. added magnetic flow as a new variable to the Butera model to explore the effects of electromagnetic induction on neuronal activities [26]. Parastesh et al. proposed a new memory function based on discontinuous flux coupling and studied various dynamic characteristics of discontinuous flux coupling neuron models [27]. Considering the mutual influence of electric and magnetic fields, Rostami and Jafari described a new Hindmarsh-Rose (HR) neuron model with more bifurcation parameters to study the formation of defects in excitable tissues and the resulting emission waves [28]. Although there have been some studies on the influence of electromagnetic fields on neuronal activity patterns, little attention has been paid to the influence of electromagnetic currents on pre-BötC.

This paper considers the effect of electromagnetic induction on the Butera neuron model with external electric current, magnetic flux, and CAN current. The membrane potential can be adjusted by a memristor that connects the membrane potential to the magnetic flux [29–32]. This paper is organised as follows. In Section 2, we describe the Butera model with a memristor. Based on this model, we explore the firing patterns of the system in Section 3. The effects of external forcing current and the magnetic flux on the bursting rhythms of the system are studied by nondimensionalization analysis, fast-slow decomposition methods, and two-parameter bifurcation analysis. The dynamical mechanisms of generation and transition of firing patterns are given. The conclusion is given in the last section.

## 2. Model Description

Electric and magnetic flow is introduced in Butera's single-compartment model of pre-BötC. The model is described as follows:

(1a) $$\begin{array}{c} \frac{\;dV}{\;dt}=\frac{-I_{\text{Na}}-I_{\text{K}}-I_{\text{L}}-I_{\text{NaP}}-I_{\text{tonic}-\text{e}}-I_{\text{CAN}}+I_{\text{extz}}-k_{1}V\rho \left(\varphi \right)}{C}, \end{array}$$

(1b) $$\begin{array}{c} \frac{dn}{dt}=\frac{n_{\infty }\left(V\right)-n}{\tau_{n}\left(V\right)}, \end{array}$$

(1c) $$\begin{array}{c} \frac{dh}{dt}=\frac{\left(h_{\infty }\left(V\right)-h\right)}{\tau_{h}\left(V\right)}, \end{array}$$

(1d) $$\begin{array}{c} \frac{\;d\varphi \;}{dt}=V-k_{2}\varphi , \end{array}$$

where *V* is the membrane potential and *n* and *h* are gating variables for the voltage-gated potassium and sodium channels, respectively. The functions *I*_Na_, *I*_K_, *I*_L_, *I*_NaP_, *I*_CAN_, and *I*_tonic−e_ represent fast sodium current, delayed rectifier potassium current, leakage current, persistent sodium current, calcium-activated nonspecific cationic current, and external tonic drive current, respectively. *I*_extz_ is a direct current of external stimuli. *C* is the whole cell capacitance. Particularly,

(2) $$\begin{array}{c} I_{\text{Na}}=g_{\text{Na}}m_{\infty }^{3}\left(V\right)\left(1-n\right)\left(V-V_{\text{Na}}\right),\\ I_{\text{K}}=g_{\text{K}}n^{4}\left(V-V_{\text{K}}\right),\\ I_{\text{NaP}}=g_{\text{NaP}}mp_{\infty }\left(V\right)h\left(V-V_{\text{Na}}\right),\\ I_{\text{L}}=g_{\text{L}}\left(V-V_{\text{L}}\right),\\ I_{\text{CAN}}=g_{\text{CAN}}f\left(\left[\text{Ca}\right]\right)\left(V-V_{\text{Na}}\right),\\ I_{\text{tonic}-\text{e}}=g_{\text{tonic}-\text{e}}\left(V-V_{\text{syn}-\text{e}}\right). \end{array}$$

The calcium dynamics is given as [13]

(3a) $$\begin{array}{c} \frac{\;d\left[\text{Ca}\right]}{dt}=K_{\text{Ca}}\left(J_{{\text{ER}}_{\text{IN}}}-J_{{\text{ER}}_{\text{OUT}}}\right), \end{array}$$

(3b) $$\begin{array}{c} \frac{\;dl}{\;dt}=AK_{d}\left(1-l\right)-A\left[\text{Ca}\right]l, \end{array}$$

where *l* represents the fraction of IP_3_ channels in the membrane of endoplasmic reticulum (ER) that have not been inactivated, which depends on the intracellular calcium concentration ([Ca]). Equation (3a) specifies that [Ca] is determined by the flux into the cytosol from the ER (*J*_ER_IN__) and the flux out of the cytosol into the ER (*J*_ER_OUT__). These fluxes are regulated by the intracellular concentration of IP_3_, [IP_3_], and IP_3_ channel gating variable, *l*.

The variable *φ* refers to the magnetic flux across the cell membrane. *ρ*(*φ*) is the coupling strength between membrane potential of neuron and magnetic flux, which is a magnetic flux-controlled memristor, and it is equivalent to memory conductance: *ρ*(*φ*) = *α* + 3*βφ*^2^, where *α* and *β* are fixed parameters [29, 30]. *k*_1_ and *k*_2_ show the relationship between the membrane potential and the magnetic flux. And we suppose that *I*_app_ = *I*_extz_ − *k*_1_*Vρ*(*φ*) in Equation (1a). The term *k*_1_*Vρ*(*φ*) introduces the inhibitory modulation of membrane potential as induction current results from variation of magnetic flux and field [25, 33], and it can be described as follows [33]:

(4) $$\begin{array}{c} \frac{dq}{dt}=\frac{dq}{d\varphi }\;\frac{d\varphi }{dt}=k_{1}V\rho \left(\varphi \right). \end{array}$$

Dynamical analysis indicates that subsystem (3a) and (3b) is in an active state only when IP_3_ lies between 0.95 *μ*M and 1.4 *μ*M [13]. Therefore, in this study, we set [IP_3_] = 1.2 *μ*M, which implies that [Ca] is not invariant. The function expressions and parameter values of other variables are presented in Appendix.

## 3. Main Results

We used the method of fast-slow decomposition to study the firing patterns of system (1a)–(3b). In order to clearly identify the timescales of different variables, we nondimensionalize the full system (1a)–(3b) as that done in previous work [34].

### 3.1. Nondimensionalization and Simplification of Timescales

The variables are rescaled so as to further reveal their timescales. To this end, we define new dimensionless variables ($v,c,\bar{\varphi },\tau$) and voltage, calcium, magnetic flux, and time scales $Q_{v},Q_{c},Q_{\bar{\varphi }}$, and *Q*_*t*_, respectively, such that

(5) $$\begin{array}{c} V=Q_{v}\cdot v,\\ \left[\text{Ca}\right]=Q_{c}\cdot c,\\ \varphi =Q_{\bar{\varphi }}\cdot \bar{\varphi },\\ t=Q_{t}\cdot \tau . \end{array}$$

Note that *n*, *h*, and *l* are already dimensionless in Equations (1b), (1c), and (3b).

Since the membrane potential *V* typically lies between −60 mV and 0 mV, we define *T*_*x*_ = max(1/*τ*_*x*_(*V*)) over the range *V* ∈ [−60, 0] and then define *t*_*x*_(*V*), a rescaled version of *τ*_*x*_(*V*), by *t*_*x*_(*V*) = *T*_*x*_*τ*_*x*_(*V*) for *x* ∈ {*n*, *h*}. As [Ca] typically lies between 0 *μ*M and 1 *μ*M, we define *G*([Ca]) = [IP_3_][Ca]/[([IP_3_] + *K*_*I*_)([Ca] + *K*_*a*_)], *g*_SERCA_([Ca]) = *V*_SERCA_([Ca]/*K*_SERCA_^2^ + [Ca]^2^) over the range [Ca] ∈ [0, 1]. Furthermore, we set *G*_*c*_ = max(*G*^3^([Ca])) and *G*_*S*_ = max(*g*_SERCA_([Ca])) and then define *P*_max_ = max{*L*_IP_3__, *P*_IP_3__*G*_*c*_, *G*_*S*_}. We also define

(6) $$\begin{array}{c} g_{\max}=\max\left\{g_{\text{N}a},g_{\text{K}},g_{\text{L}},g_{\text{NaP}},g_{\text{tonic}-\text{e}},g_{\text{CAN}},I_{\text{extz}},k_{1}\right\}. \end{array}$$

According to system (1a)–(3b), we get the following dimensionless system:

(7a) $$\begin{array}{c} \frac{C}{Q_{t}\cdot g_{\max}}\frac{\;dv}{\;d\tau }=-{\overset{¯}{g}}_{\text{Na}}m_{\infty }^{3}\left(v\right)\left(1-n\right)\left(v-{\overset{¯}{V}}_{\text{Na}}\right)-{\overset{¯}{g}}_{\text{K}}n^{4}\left(v-{\overset{¯}{V}}_{\text{K}}\right)-{\overset{¯}{g}}_{\text{L}}\left(v-{\overset{¯}{V}}_{\text{L}}\right)-{\overset{¯}{g}}_{\text{N}a\text{P}}mp_{\infty }\left(v\right)h\left(v-{\overset{¯}{V}}_{\text{Na}}\right)-{\overset{¯}{g}}_{\text{tonic}-\text{e}}\left(v-{\overset{¯}{V}}_{\text{syn}-\text{e}}\right)-{\overset{¯}{g}}_{\text{CAN}}f\left(c\right)\left(v-{\overset{¯}{V}}_{\text{Na}}\right)+{\overset{¯}{I}}_{\text{extz}}-{\overset{¯}{k}}_{1}v\rho \left(\varphi \right), \end{array}$$

(7b) $$\begin{array}{c} \frac{1}{Q_{t}\cdot T_{n}}\frac{\;dn}{\;d\tau }=\frac{n_{\infty }\left(v\right)-n}{t_{n}\left(v\right)}, \end{array}$$

(7c) $$\begin{array}{c} \frac{1}{Q_{t}\cdot T_{h}}\frac{\;dh}{\;d\tau }=\frac{h_{\infty }\left(v\right)-h}{t_{h}\left(v\right)}, \end{array}$$

(7d) $$\begin{array}{c} \frac{1}{Q_{t}}\frac{\;d\overset{¯}{\varphi }}{\;d\tau }=v-k_{2}\overset{¯}{\varphi }, \end{array}$$

(7e) $$\begin{array}{c} \frac{\sigma }{Q_{t}\cdot P_{\max}\cdot K_{\text{Ca}}}\frac{\;dc\;}{d\tau }=\left({\overset{¯}{L}}_{\text{I}{\text{P}}_{3}}+{\overset{¯}{P}}_{\text{I}{\text{P}}_{3}}G^{3}\left(c\right)l^{3}\right)\cdot \left({\left[\overset{¯}{\text{Ca}}\right]}_{\text{Tot}}-c-\sigma \cdot c\right)-{\overset{¯}{g}}_{\text{SERCA}}\left(c\right)\cdot c\cdot \sigma , \end{array}$$

(7f) $$\begin{array}{c} \frac{1}{Q_{t}\cdot Q_{c}\cdot A}\frac{\;dl}{\;d\tau }={\overset{¯}{K}}_{d}\left(1-l\right)-cl, \end{array}$$

with dimensionless parameters ${\overset{¯}{g}}_{x}=g_{x}/g_{\max}$(*x* ∈ {Na, K, L, NaP, CAN, tonic − e}), ${\overset{¯}{V}}_{x}=V_{x}/Q_{v}$(*x* ∈ {Na, K, L, syn − e}), ${\overset{¯}{I}}_{\text{extz}}=I_{\text{extz}}/\left(Q_{v}\cdot g_{\max}\right)$, ${\overset{¯}{k}}_{1}=k_{1}/g_{\max}$, $\overset{¯}{\varphi }=\varphi /Q_{\overset{¯}{\varphi }}$, ${\overset{¯}{L}}_{\text{I}{\text{P}}_{3}}=L_{\text{I}{\text{P}}_{3}}/P_{\max}$, ${\overset{¯}{P}}_{\text{I}{\text{P}}_{3}}\left(c\right)=P_{\text{I}{\text{P}}_{3}}/P_{\max}$, ${\overset{¯}{g}}_{\text{SERCA}}\left(c\right)=g_{\text{SERCA}}\left(\left[\text{Ca}\right]\right)/P_{\max}$, and ${\overset{¯}{K}}_{d}=K_{d}/Q_{c}$.

**Figure 1:**
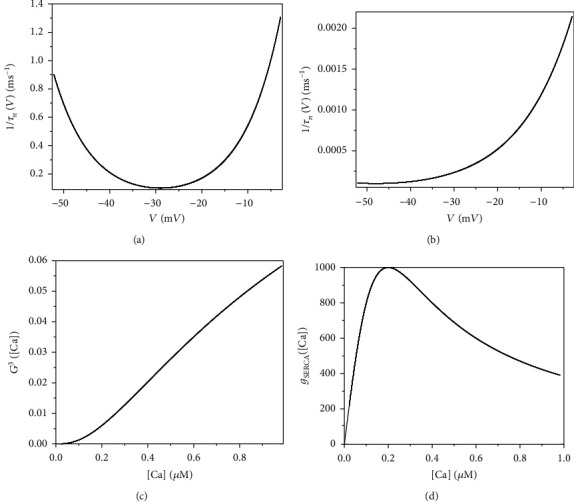
Change of functions of (a) 1/*τ*_*n*_(*V*) with membrane potential *V*, (b) 1/*τ*_*h*_(*V*) with membrane potential *V*, (c) *G*^3^([Ca]) with [Ca], and (d) *g*_SERCA_([Ca]) with [Ca].

Combining the ranges of *V*, *φ*, and [Ca], suitable choices for the voltage, magnetic flux, and calcium scales are $Q_{v}=Q_{\overset{¯}{\varphi }}=100\text{ mV}$ and *Q*_*c*_ = 1 *μ*M. We also see that values of *m*_∞_(*V*), *mp*_∞_(*V*), *n*_∞_(*V*), *h*_∞_(*V*), *f*([Ca]), *G*(Ca), ${\overset{¯}{g}}_{\text{SERCA}}\left(\left[\text{Ca}\right]\right)$, *n*, *h*, and *l* all lie in the range [0, 1]. Combining the parameter values in Table 1 and the values of variables in the paper, we have *g*_max_ = max{*I*_extz_} = 50 *μ*A. Specifically, Figures 1(a) and 1(b) show the plots of 1/*τ*_*n*_(*V*) and 1/*τ*_*h*_(*V*) over the range *V* ∈ [−60, 0], which indicates that *T*_*n*_ ≈ 1.3 ms^−1^ and *T*_*h*_ ≈ 0.0025 ms^−1^. Similarly, we obtain *G*_*c*_ ≈ 0.06 and *G*_*S*_ ≈ 1000 pL · ms^−1^ from Figures 1(c) and 1(d), respectively. So, we have *P*_max_ ≈ 1860 pL · ms^−1^. Using these values, we see that all terms in the right-hand sides of Equations (7a)–(7f) are bounded (in absolute value) by one.

The coefficients of the derivatives on the left sides of Equations (7a)–(7f) now reveal the relative rates of evolution of the variables. We find that *C*/*g*_max_ = 0.42 ms ~ *O*(1) ms, 1/*T*_*n*_ = 0.77 ms ~ *O*(1) ms, 1/*T*_*h*_ = 400 ms ~ *O*(100) ms, *σ*/(*P*_max_ · *K*_Ca_) ≈ 3.98ms ~ O(10)ms, and 1/*Q*_*c*_ · *A* = 200 ms ~ *O*(100) ms. We use the notation *O* to denote an order of magnitude estimate: *x* ~ (10^*n*^), where *n* is the nearest integer to log(*x*). We choose the fast timescale as our reference time, i.e., pick *Q*_*t*_ = 1 ms, and set

(8) $$\begin{array}{c} R_{v}≔\frac{C}{Q_{t}\cdot g_{\max}},\\ R_{n}≔\frac{1}{Q_{t}\cdot T_{n}},\\ R_{h}≔\frac{1}{Q_{t}\cdot T_{h}},\\ R_{\varphi }≔\frac{1}{Q_{t}},\\ R_{c}≔\frac{\sigma }{Q_{t}\cdot P_{\max}\cdot K_{\text{Ca}}},\\ R_{l}≔\frac{1}{Q_{t}\cdot Q_{c}\cdot A}. \end{array}$$

As a result, dimensionless system (7) becomes system (9), namely,

(9) $$\begin{array}{c} R_{v}\frac{\;dv}{\;d\tau }=-{\overset{¯}{g}}_{\text{Na}}m_{\infty }^{3}\left(v\right)\left(1-n\right)\left(v-{\overset{¯}{V}}_{\text{Na}}\right)-{\overset{¯}{g}}_{\text{K}}n^{4}\left(v-{\overset{¯}{V}}_{\text{K}}\right)-{\overset{¯}{g}}_{\text{L}}\left(v-{\overset{¯}{V}}_{\text{L}}\right)-{\overset{¯}{g}}_{\text{NaP}}mp_{\infty }\left(v\right)h\left(v-{\overset{¯}{V}}_{\text{Na}}\right)-{\overset{¯}{g}}_{\text{tonic}-\text{e}}\left(v-{\overset{¯}{V}}_{\text{syn}-\text{e}}\right)-{\overset{¯}{g}}_{\text{CAN}}f\left(c\right)\left(v-{\overset{¯}{V}}_{\text{Na}}\right)+{\overset{¯}{I}}_{\text{extz}}-{\overset{¯}{k}}_{1}v\rho \left(\varphi \right)≔f_{1}\left(v,n,h,c,I_{\text{extz}},\varphi \right),\\ R_{n}\frac{\;dn}{\;d\tau }=\frac{n_{\infty }\left(v\right)-n}{t_{n}\left(v\right)}≔\frac{f_{2}\left(v,n\right)}{t_{n}\left(v\right)},\\ R_{h}\frac{\;dh}{\;d\tau }=\frac{h_{\infty }\left(v\right)-h}{t_{h}\left(v\right)}≔\frac{f_{3}\left(v,h\right)}{t_{h}\left(v\right)},\\ R_{\varphi }\frac{\;d\overset{¯}{\varphi }}{\;d\tau }=v-k_{2}\overset{¯}{\varphi }≔f_{4}\left(v,\varphi \right),\\ R_{c}\frac{\;dc\;}{\;d\tau }=\left({\overset{¯}{L}}_{\text{I}{\text{P}}_{3}}+{\overset{¯}{P}}_{\text{I}{\text{P}}_{3}}G^{3}\left(c\right)l^{3}\right)\times \left({\left[\overset{¯}{\text{C}}\text{a}\right]}_{\text{Tot}}-c-\sigma \cdot c\right)-{\overset{¯}{g}}_{\text{SERCA}}\left(c\right)\cdot c\cdot \sigma ≔g_{1}\left(c,l\right),\\ R_{l}\frac{\;dl}{\;d\tau }={\overset{¯}{K}}_{d}\left(1-l\right)-cl≔g_{2}\left(c,l\right), \end{array}$$

with relative rates of all variables: *R*_*v*_ = *O*(1), *R*_*n*_ = *O*(1), *R*_*h*_ = *O*(100), *R*_*φ*_ = *O*(1), *R*_*c*_ = *O*(10), and *R*_*l*_ = *O*(100).

From this, we conclude that *v*, *n*, and $\overset{¯}{\varphi }$ evolve on a fast timescale, *c* evolves on a slow time scale, and *h* and *l* evolve on a super slow time scale. Thus, the above model can be regarded as a dynamic model containing three timescales: fast, slow, and super slow variables. Equations (1a), (1b), and (1d) form the fast subsystems, Equation (3a) is the slow subsystem, and Equations (1c) and (3b) form the super slow subsystem. In the current model, intracellular calcium dynamics is confined to subsystem (3a) and (3b) and evolves independently from subsystem (1a)–(1d). The dynamics of [Ca] is affected by intracellular Ca^2+^ concentration [Ca] and IP_3_ channel gating variable *l* (Equations (3a) and (3b)). For simplicity, we chose *g*_CAN_Tot__ = *g*_CAN_*f*([Ca]) as the bifurcation parameter, where *f*([Ca]) is a monotonically increasing concave function of [Ca].

According to the nondimensionalization of the model, the variable *h* is super slow. So, *h* can be considered a constant when we do the fast-slow decomposition. That is, we take *h* as the average value of the variable in the following analysis.

### 3.2. The Firing Patterns and Bifurcation Analysis without External Stimulation

**Figure 2:**
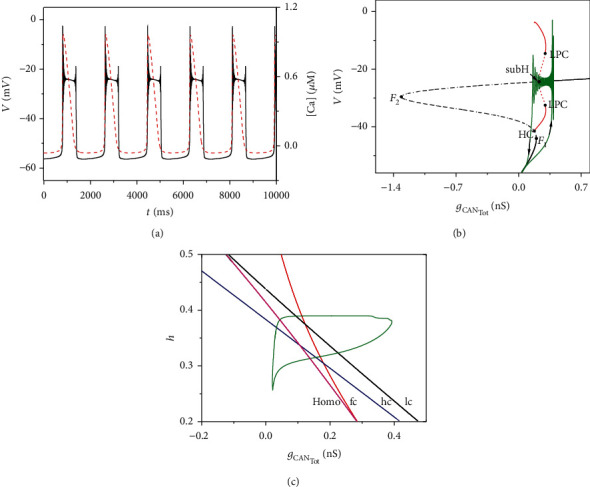
The dynamic analysis of system (1a)–(3b) without external stimulations (*I*_app_ = 0). (a) The time course of membrane potential *V* (black curve). The time course of intracellular calcium concentration [Ca] (red dashed line) is also superposed; (b) one-parameter bifurcation analysis of membrane potential; (c) two-parameter bifurcation analysis of membrane potential.

Firstly, we consider the firing activity of the full system with zero electric current and magnetic flow. Time courses of the membrane potential *V* (black solid) and the intracellular calcium concentration [Ca] (red dashed) are shown in Figure 2(a).

One-parameter and two-parameter bifurcation diagrams of the fast subsystem are shown in Figures 2(b) and 2(c), respectively. According to the nondimensionalization, the variable *h* is a super slow variable. So, *h* is considered to be a constant which we take its average value as *h* = 0.2834. *g*_CAN_Tot__ is a slow variable and regarded as a bifurcation parameter of the fast system. In this figure, the equilibrium points form an S-shaped curve. The lower (black solid line) and middle (black dash-dot line) branches of the curve are composed of the stable nodes and unstable saddles, respectively. The upper branch of the curve is composed of the stable and unstable focus separated by the subcritical Hopf bifurcation point (subH). With the decrease of *g*_CAN_Tot__, the stable focus becomes unstable, and an unstable limit cycle (red dashed line) occurs at the subcritical Hopf bifurcation (subH). The unstable limit cycle generated by the subcritical Hopf bifurcation transits into the stable limit cycle (red solid line) through the fold bifurcation of limit cycle (LPC). The points *F*_1_ and *F*_2_ refer to the fold bifurcation. The stable limit cycle disappears due to the homoclinic bifurcation (HC). The trajectory of the full system (green curve) is also appended on the bifurcation diagram. According to Izhikevich's classification scheme of bursting [35], the firing pattern of system (1a)–(3b) can be identified as “subHopf/homoclinic” bursting via “fold/homoclinic” hysteresis loop.

Figure 2(c) shows the two-parameter bifurcation analysis of the fast subsystem in (*g*_CAN_Tot__,*h*)-plane, where fc, hc, lc, and homo represent the fold bifurcation curve (red), Hopf bifurcation curve (blue), fold limit cycle bifurcation curve (black), and homoclinic bifurcation curve (purple), respectively. The trajectory of the full system curve (green) is also appended in the (*g*_CAN_Tot__,*h*)-plane. With the increase of *g*_CAN_Tot__, the trajectory passes through different bifurcation curves. Then, with the decrease of *g*_CAN_Tot__, the trajectory crosses same bifurcation curves again and drops to the origin state after meeting the homoclinic curve.

### 3.3. The Effect of Electric Current on Activity of Pre-BötC

**Figure 3:**
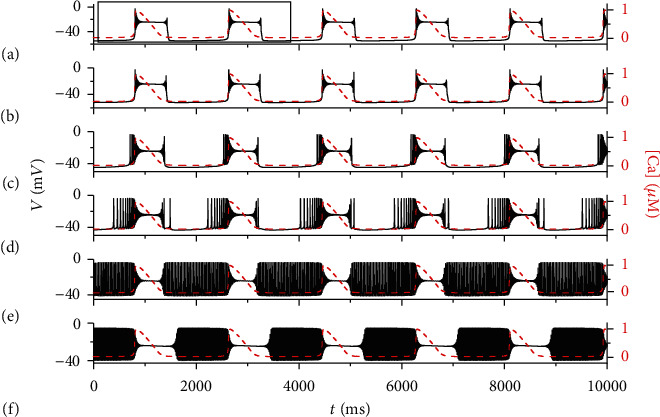
The full system (1a)–(3b) exhibits different firing patterns under different electric currents at *k*_1_ = 0.1, *k*_2_ = 3 s^−1^, *α* = 1 M*Ω*^−1^, *β* = 0.00006 M*Ω*^−1^ · V^−2^ · s^−2^. The red dashed line represents the time course of intracellular calcium concentration [Ca]. (a) *I*_extz_ = −2 *μ*A/cm^2^; (b) *I*_extz_ = 5 *μ*A/cm^2^; (c) *I*_extz_ = 25 *μ*A/cm^2^; (d) *I*_extz_ = 30 *μ*A/cm^2^; (e) *I*_extz_ = 40 *μ*A/cm^2^; (f) *I*_extz_ = 50 *μ*A/cm^2^.

Figure 3 shows some electrical activities of the system with different external forcing current *I*_extz_. For other parameter values, please refer to the appendix.

**Figure 4:**
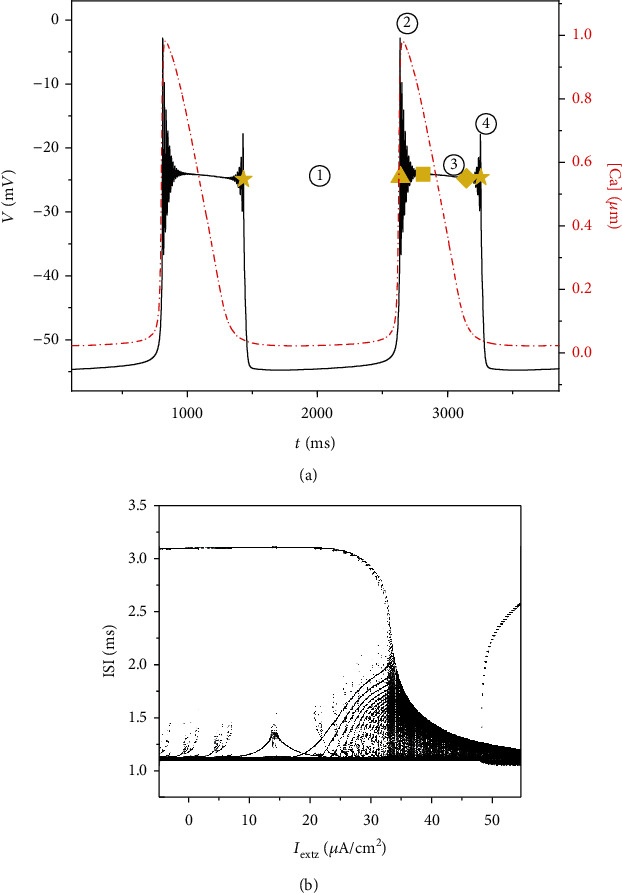
(a) Partial enlarged area of Figure 3(a). Yellow symbols mark key points along the solution trajectory (star: start of phase ①; triangle: start of phase ②; square: start of phase ③; diamond: start of phase ④); (b) the bifurcation diagram of ISI versus *I*_extz_.

The enlarged area of the two periods of Figure 3(a) is shown in Figure 4(a). We divide the change of membrane potential with time in a cycle into phases ①-④. Phase ① (from ★ to ▲) is the low potential resting stage, and its duration is about 1200 ms. Phase ② (from ▲ to ■) is tonic spikings with gradually decreasing amplitude. The time interval between adjacent spiking is very short, close to zero. Phase ③ (from ■ to ◆) is spikings with small amplitude, and the interval between spiking is very short. Phase ④ (from ◆ to ★) is spikings with gradually increasing amplitude, and the interval between adjacent spiking is also very short.

In order to further reveal the influence of external forced current on the electrical activity of neurons, the ISI (interspike interval) bifurcation diagram is drawn in Figure 4(b). We take the time interval between the maximum values of adjacent spiking as an ISI bifurcation diagram and use the logarithmic function to process the data. While the duration of phase ① increases slowly with the increases of current *I*_extz_ at first, it begins to decrease rapidly when the current value reaches 21.5 *μ*A/cm^2^. At the same time, the start time of phase ② advances, which leads to more spiking in the original low potential resting state, as shown in Figure 2 from (c) to (d). When the current increases to 33.5 *μ*A/cm^2^, the original low potential resting state disappears and all become spiking. In the process of current *I*_extz_ increase, phase ③ maintains small amplitude spikings and the duration of time gradually decreases. When the value of *I*_extz_ reaches 47.7 *μ*A/cm^2^, the original spiking of phase ③ becomes a resting state with a higher membrane potential, and then, the duration gradually increases, resulting in a gradual increase in the current value near 50 *μ*A/cm^2^ of an ISI sequence.

#### 3.3.1. Fast-Slow Decomposition

**Figure 5:**
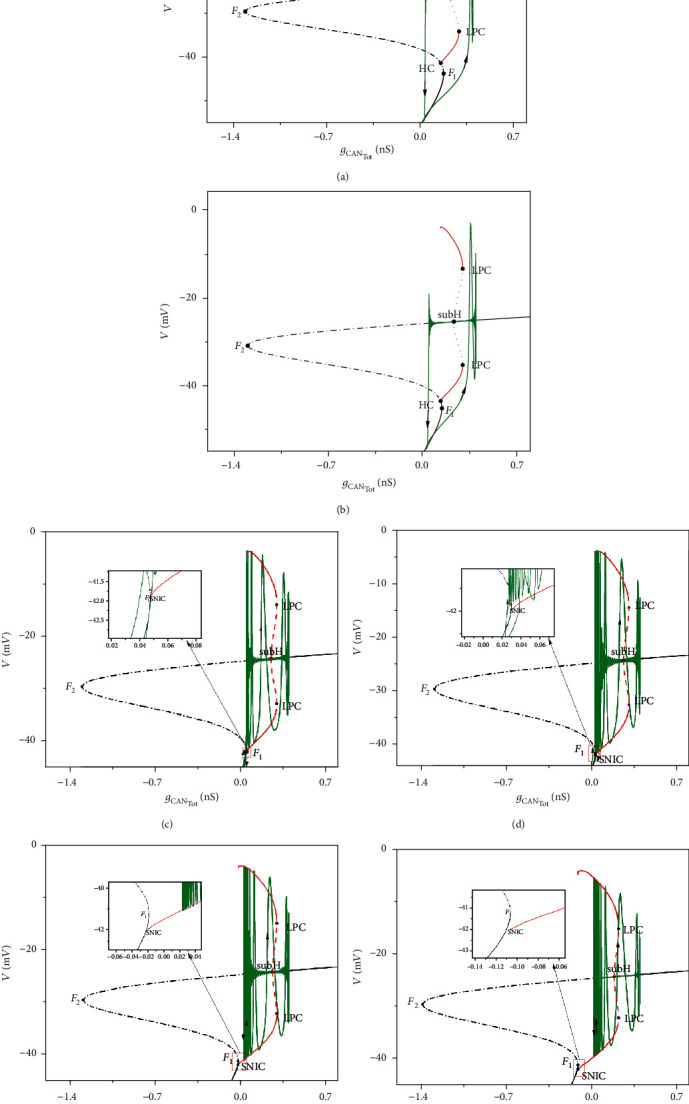
Fast-slow bifurcation analysis of neuronal firing patterns under different electric currents: (a) *I*_extz_ = −2 *μ*A/cm^2^; (b) *I*_extz_ = 5 *μ*A/cm^2^; (c) *I*_extz_ = 25 *μ*A/cm^2^; (d) *I*_extz_ = 30 *μ*A/cm^2^; (e) *I*_extz_ = 40 *μ*A/cm^2^; (f) *I*_extz_ = 50 *μ*A/cm^2^. The black solid line represents the stable nodes (lower branch) and stable focus (upper branch); the black dash-dot line represents the saddles (middle branch) and the unstable focus (upper branch); the red dashed and solid lines represent the maximum and minimum values of the unstable and stable limit cycles, respectively. The points *F*_*i*_(*i* = 1, 2) and subH represent the fold bifurcation and Hopf bifurcation of equilibrium; the points LPC, HC, and SNIC represent the fold bifurcation of the limit cycle, the homoclinic bifurcation, and saddle-node bifurcation on an invariant circle, respectively. The green curve represents the trajectory of the full system.

We can obtain one-parameter bifurcation diagrams of the fast subsystem (1a), (1b), and (1d) as shown in Figure 5, in which the projection of trajectory (the green curve) of the full system is also superposed. The equilibrium points form an S-shaped curve which is similar to that without the electric current and magnetic flow. The lower (solid black line) and middle (dash-dot line) branches of the curve are composed of stable nodes and unstable saddles, respectively. The upper branch of the curve is composed of stable and unstable focuses separated by the subcritical Hopf bifurcation point (subH). With the decrease of *g*_CAN_Tot__, the stable focuses become unstable, and the unstable limit cycles (red dashed line) occur at the subcritical Hopf bifurcation (subH) on the upper branch. The unstable limit cycles turn to be stable limit cycles (red solid circle) via fold bifurcation of limit cycle (LPC). The points *F*_1_ and *F*_2_ refer to fold bifurcation, and the point HC represents homoclinic bifurcation.

For *I*_extz_ = −2 *μ*A/cm^2^ and *I*_extz_ = 5 *μ*A/cm^2^, the bifurcation of fast subsystem (1a), (1b), and (1d) with respect to the slow variable *g*_CAN_Tot__ is shown in Figures 5(a) and 5(b), respectively. Here, we set *h* = 0.2788 and *h* = 0.2375, respectively (*h* takes the average value). Firstly, the trajectory transits from the lower rest state to the spiking state via fold bifurcation (*F*_1_). Due to the attraction of the stable focus, the amplitude of oscillation rapidly decreases until the trajectory passes through the subcritical Hopf bifurcation (subH). Then, as a result of the repelling effect of unstable focus, the amplitude of the trajectory increases gradually. Finally, the trajectory transits from spiking state to lower rest state, which completes one periodic oscillation. Thus, the firing activity of system (1a)–(3b) is the “subHopf/subHopf” bursting via “fold/homoclinic” hysteresis loop. In this case, the bistable structure is composed of the stable node on the lower branch and the stable focus on the upper branch of the equilibrium curve.

When *I*_extz_ increases to 25 *μ*A/cm^2^ and 30 *μ*A/cm^2^, the bifurcation of fast subsystem (1a), (1b), and (1d) with respect to the slow variable *g*_CAN_Tot__ is shown in Figures 5(c) and 5(d), respectively. In these two cases, the parameter *h* is set to be *h* = 0.1288 and *h* = 0.0986, respectively. The trajectory jumps up to the upper branch of the S-shaped curve via fold bifurcation *F*_1_. With the strong attraction of stable focus, the oscillation decays rapidly around the stable focus. After passing through the subcritical Hopf bifurcation point (subH), the trajectory unfolds itself progressively due to the repelling of the unstable focus and the attracting of the stable limit cycle generated by the fold limit cycle bifurcation (LPC). Finally, the trajectory drops to the lower branch of the equilibrium curve via saddle-node bifurcation on an invariant circle. The bistable structure is composed of the stable nodes on the lower branch and stable limit cycles on the upper branch. The bursting can be classified as “subHopf/fold cycle” bursting via “fold/circle” hysteresis loop.

When the value of *I*_extz_ continuously increases to 40 *μ*A/cm^2^ and 50 *μ*A/cm^2^, the bifurcation of fast subsystem (1a), (1b), and (1d) with respect to the slow variable *g*_CAN_Tot__ is shown in Figures 5(e) and 5(f), respectively. *h* is set to be *h* = 0.0490 and *h* = 0.0307, respectively. The bistable structure in this case is confirmed by the stable limit cycles and the stable focus on the upper branch of the equilibrium curve. The quiescent state is lost via subcritical Hopf bifurcation (subH), and the periodic limit cycle attractor corresponding to repetitive spiking disappears via fold limit cycle bifurcation (LPC). Hence, the bursting is “subHopf/fold cycle” bursting.

**Figure 6:**
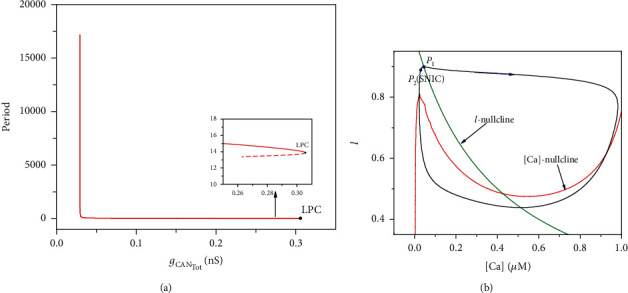
(a) The period of limit cycle of the fast subsystem with respect to parameter *g*_CAN_Tot__ at *I*_extz_ = 30 *μ*A/cm^2^; (b) the periodic orbit in subsystem (3a) and (3b). The red curve is the [Ca]-nullcline, and the green curve is *l*-nullcline. The black closed orbit is the solution of subsystem (3a) and (3b) projected in ([Ca],*l*)-space.

In Figures 5(c)–5(f), periodic orbits disappear via a saddle-node bifurcation on an invariant circle (SNIC) as *g*_CAN_Tot__ decreases. Take *I*_extz_ = 30 *μ*A/cm^2^ (Figure 5(d)) as an example, the periodic orbit of the ([Ca],*l*)-space is shown in Figures 6(a) and 6(b). Let [Ca]_SNIC_ be the value of [Ca] at SNIC; the period of the limit cycle tends to infinite when *g*_CAN_Tot__([Ca]_SNIC_) ≈ 0.02937 at which the saddle-node bifurcation on an invariant circle (SNIC) occurs. Then, we get [Ca]_SNIC_ ≈ 0.02942 according to *g*_CAN_Tot__([Ca]_SNIC_) = *g*_CAN_*f*([Ca]_SNIC_).The corresponding periodic orbit in ([Ca], *l*)-space is shown in Figure 6(b). The point *P*_1_ (blue square) denotes the time when the trajectory in subsystem (3a) and (3b) jumps up from the left knee to the right branch of the [Ca]-nullcline. Once leaving the left knee, the trajectory jumps to the right branch quickly. Thus, we have a rapid increase of [Ca] and *g*_CAN_Tot__ correspondingly, and this increase pushes the trajectory into the branch of periodic orbits. Then, the value of [Ca] slowly decreases while the trajectory in ([Ca], *l*)-space slowly moves along the right branch of the [Ca]-nullcline and finally jumps back to the left branch of [Ca]-nullcline. As long as [Ca] is greater than [Ca]_SNIC_ (*P*_2_), the system keeps firing. Once [Ca] falls below [Ca]_SNIC_, the trajectory solution stops firing and jumps down to the lower branch of the S-shaped curve (Figure 5(d)). Next, the trajectory in ([Ca], *l*)-space moves up along the left branch of the [Ca]-nullcline towards its original position, and this completes one cycle of the periodic orbit in the subsystem (3a) and (3b).

#### 3.3.2. Bifurcation Analysis

**Figure 7:**
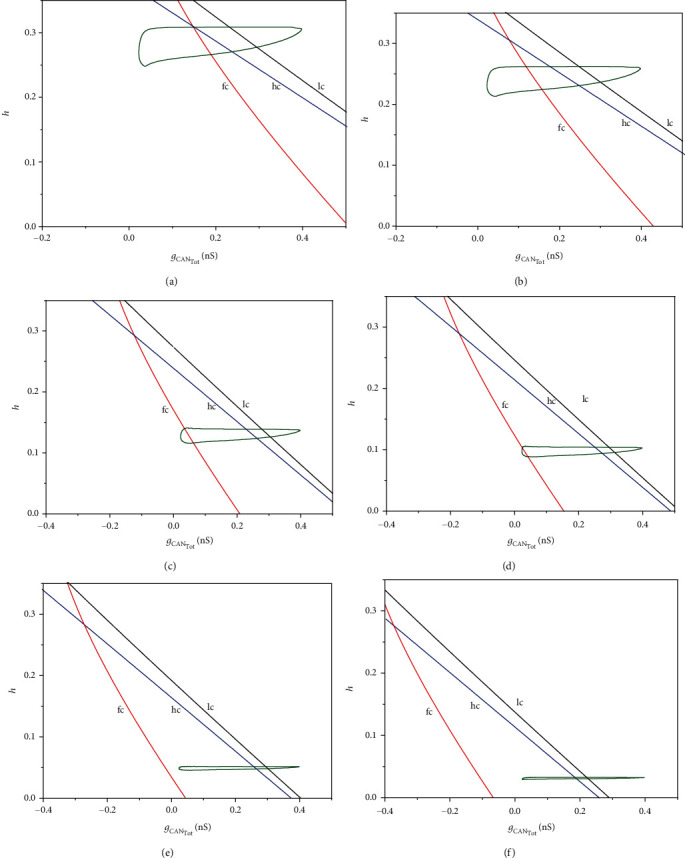
Two-parameter bifurcation diagram of the fast subsystem under the electric current in (*g*_CAN_Tot__,*h*)-plane showing the curves of fold bifurcation (fc), subcritical Hopf bifurcation (hc), and fold bifurcation of limit cycle (lc), together with projection of the trajectory of the full system (green-colored curve). (a) *I*_extz_ = −2 *μ*A/cm^2^; (b) *I*_extz_ = 5 *μ*A/cm^2^; (c) *I*_extz_ = 25 *μ*A/cm^2^; (d) *I*_extz_ = 30 *μ*A/cm^2^; (e) *I*_extz_ = 40 *μ*A/cm^2^; (f) *I*_extz_ = 50 *μ*A/cm^2^.

To better understand the mechanism of the different firing patterns evoked by the electrical current, we present two-parameter bifurcation analysis of the fast subsystem as shown in Figure 7, in which *g*_CAN_Tot__ and *h* are bifurcation parameters.

For *I*_extz_ = −2 *μ*A/cm^2^ and *I*_extz_ = 5 *μ*A/cm^2^, as shown in Figures 7(a) and 7(b), the trajectory of the full system passes through the fold bifurcation curve (fc), the Hopf bifurcation curve (hc), and the fold limit cycle bifurcation curve (lc) at the same time. The fold bifurcation curve and the Hopf bifurcation curve intersect near the full system trajectory.

When *I*_extz_ increases to 25 *μ*A/cm^2^ and 30 *μ*A/cm^2^, the trajectory of the full system moves down with the decreasing of *h*. It still passes through the three bifurcation curves of fc, hc, and lc. But the gap distance between the fold bifurcation (fc) and the Hopf bifurcation curve (hc) increases, as shown in Figures 7(c) and 7(d).

When the value of *I*_extz_ continuously increases to 40 *μ*A/cm^2^ and 50 *μ*A/cm^2^, the trajectory of the full system continues to move down to the decreasing direction of *h*. It passes through two bifurcation curves of hc and lc. The trajectory no longer passes through the fold bifurcation curve, which leads to the changes of the bistable structure, as shown in Figures 7(e) and 7(f).

The relative position of bifurcation curves and the trajectory of the system projected in (*g*_CAN_Tot__, *h*)-plane changes with the increase of *I*_extz_. That means the value of *I*_extz_ affects the critical bifurcations that determine the pattern of bursting. With the increase of *I*_extz_, the bistable structure related to bursting is changed, and the bursting with low potential resting (the stable node on the lower branch) transits to bursting with high potential resting (the stable focus on the upper branch).

### 3.4. Influence of Magnetic Flow on Activity of Pre-BötC

**Figure 8:**
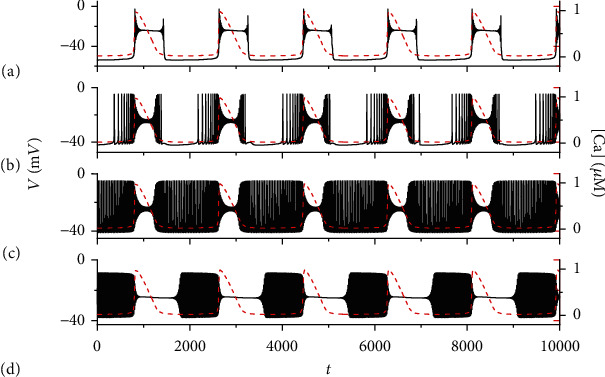
Firing pattern changes for different parameter values of *k*_1_ with *I*_extz_ = 0 *μ*A/cm^2^, *k*_2_ = 3 s^−1^, *α* = 1 M*Ω*^−1^, *β* = 0.00006 M*Ω*^−1^ · V^−2^ · s^−2^. The black solid and red dashed curves represent the time course of membrane potential *V* and intracellular calcium concentration [Ca], respectively. (a) *k*_1_ = 0.1; (b) *k*_1_ = 0.8; (c) *k*_1_ = 1.0; (d) *k*_1_ = 1.5.

We discuss the effects of magnetic flow on firing patterns of the system by switching the parameter *k*_1_. Firing activities of the system corresponding to different values of *k*_1_ are shown in Figure 8. Other parameter values are given in the appendix. The firing patterns of system (1a)–(3b) transit from one bursting pattern to another one with the external magnetic flow *k*_1_ increases.

**Figure 9:**
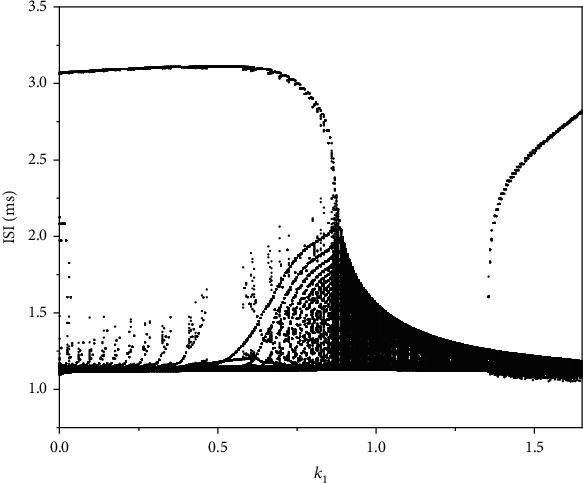
The bifurcation diagram of ISI versus *k*_1_.

The ISI bifurcation diagram of the magnetic flow feedback coefficient *k*_1_ is shown in Figure 9. Circled numbers are the same as that in Figure 4(a). As *k*_1_ increases, the duration of phase ① first slowly increases and then decreases. When *k*_1_ increases to 0.88, the original low potential resting state turns to be spiking. With the increase of *k*_1_, phase ③ exhibits small amplitude oscillating, and its duration gradually decreases. When the value of *k*_1_ reaches 1.33, the small amplitude oscillating in phase ③ becomes a resting state with a higher membrane potential, and at the same time, its duration gradually increases, which results in a gradual increase of ISI sequence.

**Figure 10:**
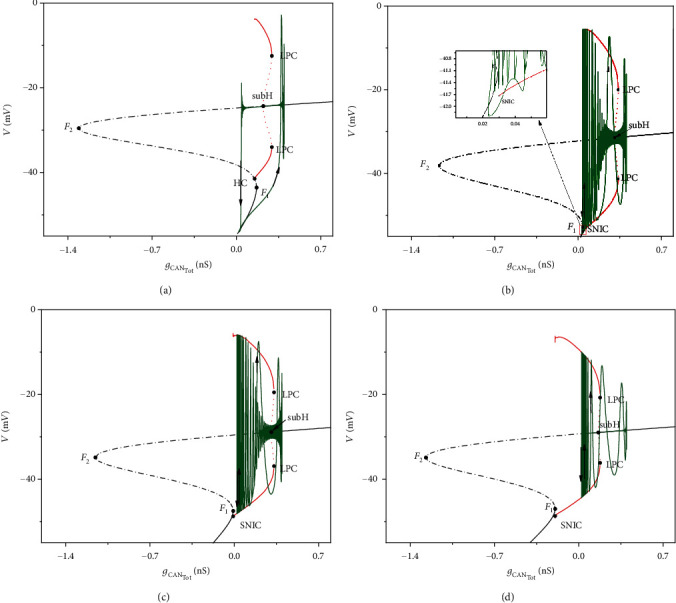
Fast-slow bifurcation analysis of neuronal firing patterns with different magnetic flow for (a) *k*_1_ = 0.1; (b) *k*_1_ = 0.8; (c) *k*_1_ = 1.0; (d) *k*_1_ = 1.5.

Similarly, Equations (1a), (1b), and (1d) form the fast subsystem, and [Ca] is still the slow variable. We use *g*_CAN_Tot__ = *g*_CAN_*f*([Ca]), instead of [Ca], as the bifurcation parameter, and set *h* to be constant. The bifurcation diagrams of the fast subsystem on the (*g*_CAN_Tot__,*V*)-plane are shown in Figure 10. With the parameter *k*_1_ increasing, the bistable structure also changes. The bursting with low potential resting (the stable node on the lower branch shown in Figures 10(a) and 10(b)) transits to bursting with high potential resting (the stable focus on the upper branch shown in (Figures 10(c) and 10(d)). In (Figures 10(b)–10(d)), periodic orbits also disappear via a saddle-node on an invariant circle (SNIC) bifurcation as *g*_CAN_Tot__ decreases. The analysis is similar to that in Figure 6.

**Figure 11:**
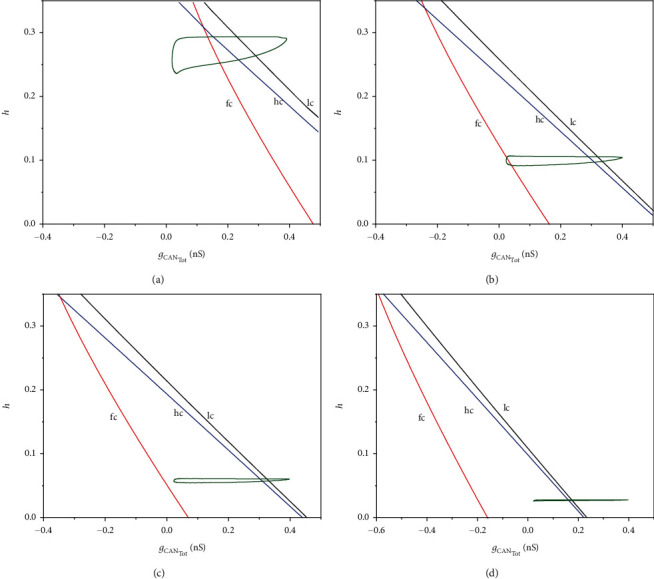
Two-parameter bifurcation diagram with the effect of magnetic flow on (*g*_CAN_Tot__,*h*)-plane for (a) *k*_1_ = 0.1; (b) *k*_1_ = 0.8; (c) *k*_1_ = 1.0; (d) *k*_1_ = 1.5.

Two-parameter bifurcation of the fast subsystem in (*g*_CAN_Tot__,*h*)-plane is shown in Figure 11. Similarly, with the increase of *k*_1_, the distance between the curves of fold bifurcation (hc) and Hopf bifurcation (lc) increases, which results in the changes of the bistable structure. The parameter *k*_1_ and the electric current have the same effect on the bursting transition.

**Figure 12:**
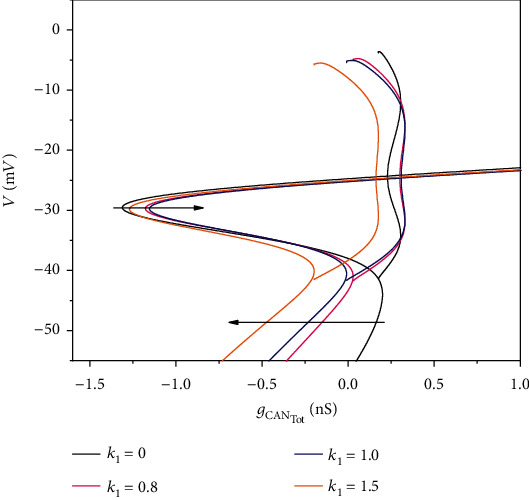
The effect of *k*_1_ on the bifurcation diagram. The black curve is the bifurcation curve when *k*_1_ = 0 and *I*_extz_ = 0, that is, the bifurcation curve when there is no external stimulus. The color curves are the bifurcation curves with a nonzero external stimulus. Corresponding values of *k*_1_ are marked in the upper right corner of the figure.

With the increase of *k*_1_, the middle branch of the S-shaped curve moves to the right and the lower branch to the left, as shown in Figure 12. Compared with the case when there is no magnetic flow stimulus, the increase of *k*_1_ does not change the bifurcation structure but changes the position of the bifurcation point.

**Figure 13:**
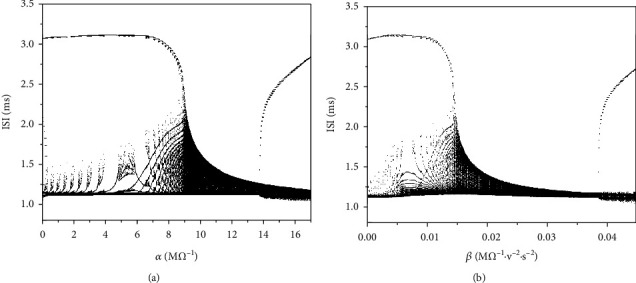
(a) The bifurcation diagram of ISI versus *α*; (b) the bifurcation diagram of ISI versus *β*.

Finally, we give the ISI bifurcation diagram of *α* and *β* to illustrate the influence of the magnetic flow on firing patterns, as shown in Figures 13(a) and 13(b), respectively. The bifurcation structure of ISI is similar to that of Figures 4(b) and 9, which indicates that as the value of *α* or *β* increases, the shape of the bursting will change. When the value of *α* or *β* is large enough, the bursting will transit from low potential resting to high potential resting at different threshold values. Increasing the value of *α* or *β* can accelerate the transition from low-potential resting state to high-potential resting state.

## 4. Conclusion

Bifurcation is one of the most important tools for understanding the generation and transition of rhythms. Changes in system parameters or external stimulations lead to the appearance of different bifurcations. Based on Butera's model with a memristor, the effects of electric current and magnetic flow on firing patterns of the system are studied by means of fast-slow decomposition and bifurcation analysis. We found that both the direct current and magnetic flux affect the rhythm of the pre-BötC neuron significantly.

The structure of two-parameter bifurcation gives us much information about the pattern of firing. The direct current *I*_extz_ can affect the relative position of the bifurcation curves, which will lead to changes of the bistable structure of the fast subsystem. The stability composed of the stable nodes on the lower branch and stable focus on the upper branch changes to the stability composed of stable focus and stable limit cycle on the upper branch with the increase of *I*_extz_, which indicates that the control of direct current *I*_extz_ can lead to desired firing patterns of the system. The variation of magnetic flow can result in a similar effect. The results in this study may help to reveal and explain the dynamic mechanism of electromagnetic pathogenesis and may provide theoretical guidance to diagnosing the diseases with pathological respiratory rhythm.

## Figures and Tables

**Table 1:** Parameter values used in the paper.

Parameter	Value	Parameter	Value	Parameter	Value
*C*	21 *μ*F	*θ*_*n*_	−29 mV	*K*_CAN_	0.74 *μ*M
*σ*	0.185	*θ*_*h*_	−48 mV	*n*_CAN_	0.97
*g*_L_	2.8 nS	*θ*_*m*_	−34 mV	[IP_3_]	1.2 *μ*M
*g*_K_	4 nS	*θ*_*mp*_	−40 mV	*L*_IP_3__	0.37 pL·s^−1^
*g*_Na_	10 nS	*σ*_*n*_	−4 mV	*P*_IP_3__	31,000 pL·s^−1^
*g*_NaP_	2.8 nS	*σ*_*h*_	6 mV	[Ca]_Tot_	1.25 *μ*M
*g*_tonic−e_	0.3 nS	*σ*_*m*_	−5 mV	*K*_Ca_	0.000025 pL^−1^
*g*_can_	0.7 nS	*σ*_*mp*_	−6 mV	*V*_SERCA_	400 aMol·s^−1^
*V*_Na_	50 mV	${\overset{¯}{\tau }}_{n}$	10 ms	*K*_ERCA_	0.2 *μ*M
*V*_K_	−85 mV	${\overset{¯}{\tau }}_{h}$	10,000 ms	*A*	0.005 *μ*M·s^−1^
*V*_L_	−65 mV	*K*_*I*_	1.0 *μ*M	*K*_*d*_	0.4 *μ*M
*V*_syn−e_	0 mV	*K*_*a*_	0.4 *μ*M		

## Data Availability

The data used to support the findings of this study are available from the corresponding author upon request.

## Appendix

For *x* ∈ {*n*, *h*, *m*, *mp*}, the function of *x*_∞_(*V*) takes the form *x*_∞_(*V*) = 1/(1 + exp((*V* − *θ*_*x*_)/*σ*_*x*_)).

For *x* ∈ {*n*, *h*}, the function of *τ*_*x*_(*V*) takes the form $\tau_{x}\left(V\right)={\overset{¯}{\tau }}_{x}/\cosh\left[\left(V-\theta_{x}\right)/\left(2\sigma_{x}\right)\right]$.

The activation of the CAN current by the calcium concentration is given as

(A.1) $$\begin{array}{c} f\left(\left[\text{Ca}\right]\right)=\frac{1}{1+{\left(K_{\text{CAN}}/\left[\text{Ca}\right]\right)}^{n_{\text{CAN}}}}. \end{array}$$

[IP_3_], as well as *l*, is described as follows:

(A.2) $$\begin{array}{c} J_{{\text{ER}}_{\text{IN}}}=\left(L_{{\text{IP}}_{3}}+P_{{\text{IP}}_{3}}{\left[\frac{\left[{\text{IP}}_{3}\right]\left[\text{Ca}\right]l}{\left(\left[{\text{IP}}_{3}\right]+K_{I}\right)\left(\left[\text{Ca}\right]+K_{a}\right)}\right]}^{3}\right)\times \left({\left[\text{Ca}\right]}_{\text{ER}}-\left[\text{Ca}\right]\right),\\ J_{{\text{ER}}_{\text{OUT}}}=V_{\text{SERCA}}\left(\frac{{\left[\text{Ca}\right]}^{2}}{K_{\text{SERCA}}^{2}+{\left[\text{Ca}\right]}^{2}}\right), \end{array}$$

in which [Ca]_ER_ is given as

(A.3) $$\begin{array}{c} {\left[\text{Ca}\right]}_{\text{ER}}=\frac{{\left[\text{Ca}\right]}_{\text{Tot}}-\left[\text{Ca}\right]}{\sigma }. \end{array}$$
